# Delayed dispersal and the costs and benefits of different routes to independent breeding in a cooperatively breeding bird

**DOI:** 10.1111/evo.13071

**Published:** 2016-10-06

**Authors:** Sjouke A. Kingma, Kat Bebbington, Martijn Hammers, David S. Richardson, Jan Komdeur

**Affiliations:** ^1^ Behavioural & Physiological Ecology, Groningen Institute for Evolutionary Life SciencesUniversity of Groningen9700CCGroningenThe Netherlands; ^2^ Centre for Ecology, Evolution and Conservation, School of Biological SciencesUniversity of East AngliaNorwichNR4 7TJUnited Kingdom; ^3^ Nature SeychellesMahéSeychelles

**Keywords:** Benefits‐of‐philopatry, cooperative breeding, delayed dispersal, ecological constraints, informed dispersal, reproductive skew

## Abstract

Why sexually mature individuals stay in groups as nonreproductive subordinates is central to the evolution of sociality and cooperative breeding. To understand such delayed dispersal, its costs and benefits need to be compared with those of permanently leaving to float through the population. However, comprehensive comparisons, especially regarding differences in future breeding opportunities, are rare. Moreover, extraterritorial prospecting by philopatric individuals has generally been ignored, even though the factors underlying this route to independent breeding may differ from those of strict philopatry or floating. We use a comprehensive predictive framework to explore how various costs, benefits and intrinsic, environmental and social factors explain philopatry, prospecting, and floating in Seychelles warblers (*Acrocephalus sechellensis*). Not only floaters more likely obtained an independent breeding position before the next season than strictly philopatric individuals, but also suffered higher mortality. Prospecting yielded similar benefits to floating but lower mortality costs, suggesting that it is overall more beneficial than floating and strict philopatry. While prospecting is probably individual‐driven, although limited by resource availability, floating likely results from eviction by unrelated breeders. Such differences in proximate and ultimate factors underlying each route to independent breeding highlight the need for simultaneous consideration when studying the evolution of delayed dispersal.

In many vertebrate species, sexually mature individuals delay dispersal and independent breeding and stay as philopatric subordinates in a group (Taborsky 1994; Clutton‐Brock and Lukas 2012; Drobniak et al. 2015). Given that evolutionary theory predicts that individuals should maximize their own fitness, why individuals employ this strategy is central to our understanding of the evolution of group living and cooperative breeding (Wiley and Rabenold 1984; Koenig et al. 1992; Hatchwell 2009). Two complementary hypotheses have been proposed to explain delayed dispersal (Koenig et al. 1992; see Table 1). The “ecological‐constraints” hypothesis (Selander 1964; Emlen 1982) predicts that individuals cannot acquire an independent breeding position when suitable breeding vacancies are limited (e.g., due to habitat saturation) or too costly to find. Second, the “benefits‐of‐philopatry” hypothesis (Stacey and Ligon 1987) predicts that individuals intentionally delay dispersal when the survival and/or reproductive fitness benefits of remaining as a subordinate in a territory exceed those of leaving (Kokko and Ekman 2002; Griesser et al. 2006; Kingma et al. 2011, 2014). Based on these hypotheses, several studies have attempted to determine the costs, benefits, and constraints of delayed dispersal. However, there is no consensus about which factors are the most important drivers of delayed dispersal (Hatchwell and Komdeur 2000; Ekman et al. 2004; Ekman 2006; Hatchwell 2009). This hinders our understanding of the evolution of delayed dispersal, and, therefore, of how complex social systems like cooperative breeding remain evolutionarily stable.

**Table 1:** Summary of the hypotheses and evolutionary concepts relating to how the relative survival and reproductive benefits of dispersal and philopatry may drive prospecting and floating behavior and, ultimately, cooperative behavior or sociality in vertebrates

				Prediction met in Seychelles warblers		
Theory	Principles	Concepts	Predictions relative to philopatry in Seychelles warblers	Prospectors	Floaters	Residents
Benefits of prospecting and floating	Survival benefits: access to food	1. Better access to food outside resident territory	1. Prospectors/floaters are in better condition	No	No	
			2. Prospectors/floaters have better access to food	No	No	
	Reproductive benefits: improved—current or future—reproduction	2A. Higher likelihood of obtaining a territory	1. Prospectors/floaters are more likely to obtain a territory	Yes	Yes	
			2. Prospectors/floaters get a territory further away	Yes	Yes	
		2B. Obtaining a better quality breeding position	1. Prospectors/floaters obtain a territory with higher food abundance	No	No	
			2. Prospectors/floaters get a less related partner	Yes	No	
		2C. Access to extrapair mating	1. Prospecting/floating males fertilize extragroup females	No^1^	No^1^	
			2. Prospecting/floating females are fertilized by an extragroup male	Maybe^1^	No^1^	
			3. Females (mainly) prospect in fertile period	No	N/A	
Ecological constraint	Habitat saturation	3. No suitable breeding habitat for independent breeding available	1. Individuals that do not find a vacancy return to resident territory	Yes	No	
			2. Vacancies are filled very rapidly	Yes/No^2^	Yes/No^2^	
			3. Breeding positions are obtained by inheritance only/mainly	No^3^	No^3^	
Ecological constraints and	Survival benefits: a resident territory	4A. Reduced predation	1. Prospectors/floaters suffer more predation	No	No	
survival benefits‐of‐philopatry	provides a “safe‐haven”	4B. Access to food in resident territory	1. Individuals are tolerated in resident territory	Yes	No	
			2. Individuals in good quality resident territories are less likely to leave	No	No	
			3. Related individuals prospect/float less (nepotism)	No	Yes	
			4. Individuals in smaller groups prospect/float less	No	Yes	
		4C. Prospecting is energetically costly	1. Prospectors/floaters are in worse condition	Yes	No^4^	
			2. Prospectors/floaters have lower survival	No^4^	Yes	
			3. Prospectors/floaters are attacked in intruded territory	Yes	Yes	
			4. Prospecting/floating is condition dependent	Maybe^4^	N/A	
Reproductive benefits‐of‐philopatry	Reproductive benefits: individuals stay in a resident territory for—future, current, or indirect—reproductive benefits	5A. Territory inheritance	1. Resident individuals inherit the territory	No^5^	N/A	Only 8%^5^
		5B. Access to nearby vacancies ( = *shifting*)	1. Individuals obtain a breeding position nearby resident territory	Yes^5^	N/A	Yes^5^
			2. Individuals with more neighboring territories prospect/float less	No	No	
			3. Competitive individuals prospect/float less	No/Yes^6^	No^6^	
		5C. Access to own reproduction	1. Individuals obtain parentage in their resident territory	Yes^7^	N/A	Yes^7^
			2. Individuals prospect/float less if opposite‐sex breeder is unrelated	No	N/A	
			3. Females prospect/float less than males (more parentage^7^)	No	No	
		5D. Individuals can obtain (indirect) benefits from helping	1. Nonhelpers prospect more than helpers	No	N/A	
			2. Individuals related to the breeders prospect/float less (kin benefits)	No	Yes	

To test whether these concepts may explain subordinate behavior in Seychelles warblers, a set of specific predictions was developed and tested in this study. Prospectors were individuals with a resident territory who made temporary prospecting trips, whereas floaters left a territory permanently to float through the population with no resident territory (note therefore that some predictions only apply to prospectors as specific data are not available for floaters (N/A).

^1^Subordinate females sometimes lay eggs sired by an extragroup male (Richardson et al. 2001). It is unclear, however, whether these fertilizations are a result of male pursuits, or females actively prospecting to obtain copulations with extragroup males (although most females prospected outside their fertile period [see Results] suggesting that the primary reason for females to prospect is not extragroup matings). Moreover, extragroup offspring are always sired by dominant males, and egg‐dumping (e.g., by floaters) is absent (Richardson et al. 2001).

^2^Eikenaar et al. (2009) showed that the median time to occupation of a breeding vacancy was 3.5 days, with a range of 1–20 days. Two vacancies remained unoccupied for more than 3 months.

^3^No direct evidence, but tendency for individuals in better quality territories being more likely to prospect (*P* = 0.06–0.08; Table 4) and prospecting seems to occur mainly in periods with high food availability (Supporting Information).

^4^Nonsignificant tendency (see Fig. 1).

^5^Of the 170 individuals that obtained a breeding position, 81 (48%) obtained a position further than two territories away from their last resident territory, 75 (44%) obtained a territory nearby (one or two territories from their last resident territory), and 14 (8%) inherited the resident territory.

^6^Males prospect less than females, but prospecting is independent of age (Table 4); both sexes, however, may be considered “competitive” as both defend a territory. Floating was independent of age and sex.

^7^Forty‐four percent of subordinate females lay an egg, but only one of 55 young was sired by a subordinate male (Richardson et al. 2001, 2002). Subordinate cobreeding females are, however, almost always older (>1 year) subordinates (Kingma et al., unpubl. ms.).

To determine the costs and benefits of delayed dispersal, one must compare the survival and reproductive benefits of philopatry with those of dispersing to an independent breeding position. For individuals that postpone independent breeding, the most obvious alternative to passively waiting for a local position is “floating,” whereby individuals permanently leave their resident territory to actively search for an independent breeding position in the wider population (Ridley et al. 2008). Therefore, to understand delayed dispersal in group‐living species it is important to consider why individuals do not float, rather than why they postpone independent breeding (Koenig et al. 1992; Ridley et al. 2008). This question essentially addresses the balance between the costs and benefits of delayed dispersal versus those of floating. It is important since floaters may improve their chances of finding a breeding vacancy (Brown 1987; Zack and Stutchbury 1992; Kokko and Ekman 2002) or a better quality territory (Koenig et al. 1992) compared to exclusively philopatric individuals who are restricted to obtaining a breeding position in or near the natal territory (Woolfenden and Fitzpatrick 1978; Zack 1990; Komdeur and Edelaar 2001; Kokko and Ekman 2002). One potential answer is that floating through unfamiliar terrain, with limited possibilities for refuge, may lead to attacks by conspecifics or predators and subsequent energetic and survival costs (e.g., Griesser et al. 2006; Ridley et al. 2008; Young and Monfort 2009). However, the costs and benefits of floating have received limited empirical attention (Zack 1990; Ridley et al. 2008), probably because they are difficult to study (e.g., floaters may be difficult to monitor and follow, especially in open study populations). Therefore, it remains unclear whether floaters have better access to vacancies than philopatric individuals (Smith and Arcese 1989; Zack 1990; Koenig et al. 1992; Ekman et al. 2004).

Despite its potentially far‐reaching importance (Koenig et al. 1992), studies determining the benefits of philopatry relative to floating have often ignored that, in addition to passively waiting, philopatric individuals may “prospect” as an active tactic to find an independent breeding position. This is somewhat surprising because in many social species (in which breeding vacancies are limitedly available) philopatric subordinates assess the availability and quality of breeding territories over a large area through temporary prospecting trips outside their resident territory (Reed et al. 1999; Young and Monfort 2009). Theoretical studies of prospecting (also referred to as “stay‐and‐foray”) assume that such assessments are limited to territories near the prospectors’ resident territory (Brown 1987; Koenig et al. 1992; Kokko and Ekman 2002), but it remains unclear whether this is actually the case. In fact, prospecting may yield similar reproductive benefits as floating (e.g., assessing territory availability and quality over relatively large areas), but, unlike floaters, prospectors may also obtain the benefits of philopatry by returning home after prospecting trips. A detailed evaluation of the relative long‐term benefits of prospecting while philopatric (hereafter shortened to “prospecting”) in comparison with floating and strict philopatry is missing, but could provide new insights into the costs and benefits of different dispersal strategies.

Simultaneously considering different routes to an independent breeding position (i.e., philopatry, floating, and prospecting) is key to achieving a comprehensive understanding of the proximate and ultimate factors that maintain delayed dispersal for two main reasons (Pasinelli and Walters 2002; Mares et al. 2014). First, as mentioned above, the costs and benefits of different routes may differ (Table 1) and this will determine the relative cost‐benefit ratio of delayed dispersal (Koenig et al. 1992; Komdeur 1992). Second, considering different routes to independent breeding have important implications for studying the proximate environmental (e.g., spatial and temporal variation in food availability), social (e.g., acceptance by breeders, group size) and intrinsic (e.g., sex, age, and condition) factors that may underlie delayed dispersal, because these factors may differentially affect the costs and benefits of the different routes. That said, so far remarkably little is known about these routes and the proximate factors that may determine individual decisions (Pasinelli and Walters 2002; Ridley et al. 2008; Ponchon et al. 2013; Mares et al. 2014).

In this study we simultaneously assess the potential costs and benefits of the different routes to independent breeding described above, as well as the proximate factors underlying them (see Table 1 for a comprehensive overview). We use this framework to generate predictions about delayed dispersal in the cooperatively breeding Seychelles warbler (*Acrocephalus sechellensis*). Specifically, we (1) explore the relative costs and benefits of philopatry, prospecting, and floating and (2) determine the environmental, social, and intrinsic proximate factors that may drive these behaviors; to (3) make inferences about the value of distinguishing these routes to independent breeding for our understanding of the evolution of delayed dispersal.

As a result of habitat saturation, breeding vacancies in the Seychelles warbler population are limitedly available. As a probable consequence, subordinate Seychelles warblers often delay independent breeding and remain in their resident territory beyond sexual maturity (Komdeur 1992). This system has several advantages for studying routes to independent breeding: (1) several routes to breeding occur in this system (Komdeur and Edelaar 2001; Eikenaar et al. 2008): subordinates can obtain a breeding position through inheritance, shifting to a neighboring territory, prospecting (relatively brief extraterritorial forays after which, if unsuccessful at finding a vacancy, individuals return to their resident territory; see Kingma et al. 2016), or floating through the population without a resident territory. (2) Our study population lives on a small saturated island from which emigration is virtually absent (Komdeur et al. 2004b) so all individuals can be studied and mortality data are not confounded by dispersal from the field site (see Koenig et al. 1996). (3) The detailed long‐term nature of this study (providing accurate data on behavior, survival rates, relatedness between individuals, and temporal and spatial variation in food availability) allows us to identify prospectors and floaters, to determine the proximate factors of dispersal, and to assess whether these behaviors yield benefits such as access to extragroup fertilizations or food availability (e.g., Young et al. 2005; Kesler and Haig 2007; Mares et al. 2014). (4) Prospecting individuals that do not obtain a breeding position often return to their resident territory (Eikenaar et al. 2008; also see Results), so we can exclude that prospecting individuals were evicted from their territory. (5) Adult Seychelles warblers have no predators, so the potential survival costs of prospecting and floating are most likely due to reduced food intake and intraspecific aggression (Kingma et al. 2016). This also allows us to test the effect of food availability in the resident territory on delayed dispersal.

We first test whether prospecting and floating help subordinates to obtain a territory, rather than provide access to food or extragroup fertilization. Second, we make inferences about the relative fitness benefits of prospecting and floating by assessing whether individuals that apply either of these behaviors have a higher likelihood of obtaining a breeding position (which are limited and arise stochastically as a result of breeder disappearance and the possibility to reproduce is therefore likely a strong determinant of fitness in this species) or a better quality territory (Table 1) than strictly philopatric individuals. Third, we use our predictive framework (Table 1) to assess the cost and benefits of the different routes to breeding, and to investigate whether a suite of potential proximate factors predicts whether individuals remain strictly philopatric, prospect, or float (see Methods and Table 1 for specific predictions). Altogether, this comprehensive analysis aims to provide insight into the role of individual, ecological, and social factors that determine the costs and benefits of different routes to independent breeding.

## Materials and Methods

### STUDY SYSTEM AND FIELDWORK

We studied an isolated population of about 320 individually color‐ringed Seychelles warblers in about 110 territories on Cousin Island, Seychelles (29 ha; 04°20′S, 55°40′E) from 2003 until 2014. The birds form small groups in territories where approximately 50% of breeding pairs are accompanied by one to four independent subordinates (average ± SE number of subordinates per territory = 0.7 ± 0.02; *n* = 1385 group years). Subordinates can be of either sex (55% of the 390 subordinates considered here were female), and are usually retained offspring of the breeding pair (69% of 390 individuals), but can also be unrelated to one or both breeders (13% and 18%, respectively). Territories are stable year‐round (breeders usually remain present until death; Hammers et al. 2015) and are defended against intruding conspecifics (who are physically attacked; Kingma et al. 2016). Territory boundaries are easily determined based on the movement and foraging behavior of the birds and border disputes between groups. Approximately half of all subordinate individuals engage in helping behavior in any given breeding season (incubation, nestling feeding, and territory defense; Komdeur 1994). Breeding vacancies, which arise following breeder mortality, are limited as Seychelles warblers live up to 18 years (average life span from fledging = 5.5 year; Komdeur 1991; Hammers et al. 2015) and all suitable habitat on the island is occupied (Komdeur 1992; Komdeur et al. 2016). As breeder dispersal is rare, vacancies are usually filled by subordinates from the same or other territories, or by floaters (Eikenaar et al. 2009). Despite the presence of a surplus of nonbreeding subordinates, breeding vacancies can remain unoccupied relatively long (median: 3.5 days, range: 1–20 days; Eikenaar et al. 2009).

Breeding takes place year‐round, but mainly in the “main breeding season” (June–September) or sometimes in the “minor breeding season” (December–January or February). Because the duration of this fieldwork period in the minor breeding season is brief (so prospectors and floaters are easily missed), we use only data from the main breeding season (average ± SE duration = 94 ± 5 days, range = 59–110, *n* = 12). Each season, as many birds as possible were captured using mist nets (see Kingma et al. 2016 for details about the catching protocol) and, if not already ringed, given a unique combination of three color rings and a numbered metal ring. We took a small blood sample from each bird for genetic analyses (to confirm sex and estimate relatedness), and measured body mass (±0.1 g) and tarsus length (±0.1 mm). We performed regular censuses to determine all individuals’ resident territory (i.e., where birds were consistently observed feeding and involved in nonantagonistic interactions with other resident individuals) and breeding status (breeder, subordinate, or independent juveniles; the latter 3–5 months old, as assessed by gray eye color; Komdeur 1991). In territories with nests, we determined whether subordinates engaged in helping (incubation and/or feeding nestlings) by performing at least one incubation watch and/or feeding watch of 1 hour, which is long enough to reliably assess helping status (Komdeur 1994; Van de Crommenacker et al. 2011a). Seychelles warblers are insectivorous and territory quality is measured based on monthly assessments of arthropod prey availability within each territory following Komdeur (1992) and Van de Crommenacker et al. (2011b). In one season territory quality data were not collected, and we used the average of the preceding and following seasons (Brouwer et al. 2006). Exclusion of this season did not change the results (not shown).

### STATUS ASSIGNMENTS: PHILOPATRIC INDIVIDUALS, PROSPECTORS, AND FLOATERS

Each season, all subordinates were assigned a status (philopatric, prospector, or floater) based on where they were observed and caught throughout that season. We determined the minimal distance (in number of territories) between the location of any observations/catches and the individual's resident territory. Birds only observed or caught within two territories of their resident territory were classified as “strictly philopatric individuals.” This classification criterion was used to reduce the likelihood of assigning philopatric individuals as prospectors when they were merely being attracted to song playback (used to help capture birds) or just intruding into nearby territories (Kingma et al. 2016). Birds were usually observed in their resident territory but who at some point in the season were observed or caught more than two territories away from their resident territories were classified as *prospectors* (Kingma et al. 2016). These birds were (unless observed prospecting at the end of the fieldwork period; 12 out of 54) observed again back in their territory after relatively short prospecting trips (see Results and Kingma et al. 2016 for details). Birds that were only observed on nonresident territories throughout the season (i.e., without a resident territory) were classified as *floaters* (each floater was observed in on average (±SE) 2.81 ± 0.24 territories per season; median = 3, *n* = 38, range = 1–7).

### STATISTICAL ANALYSES

All analyses were performed using the *lme4* package (version 1.1‐8; Bates et al. 2014) in R 3.2.0 (R development core team 2015) using general (for normally distributed response data) or generalized (for binomial and Poisson data) linear mixed models, including year as random effect. Nonsignificant variables (*P* > 0.05) were sequentially excluded, starting with the least significant variable, until the model only contained significant variables. Nonsignificant variables were checked by reincluding these in the final model, and the values after inclusion are reported. Average values and model estimates (β) are reported ± standard error (SE).

For analyses including subordinate “status” (i.e., philopatric, prospector, floater) we only included individuals that were already ringed or caught within the first month of the season to avoid falsely assigned birds that had prospected while unringed and unidentifiable. Subordinates demoted from a breeder position (Richardson et al. 2007) were not included. Independent juveniles (3–5 months old) rarely prospected or floated (5% and 2% of 131 individuals, respectively) and subordinates older than 2 years (*n* = 119) were never observed prospecting or floating, so these were not included in analyses. We classified the remaining birds as subadult (>5 months–1 year) or adult (older than a year) subordinates. Unless stated otherwise, we used only the first recorded status or catch of each individual in analyses of dispersal and survival to avoid pseudoreplication.

#### Benefits of prospecting and floating: territory acquisition and extragroup fertilizations

Subordinates may potentially prospect or float to obtain reproductive benefits (Concept 2 in Table 1), including: (1) a higher likelihood of obtaining a territory, (2) a better quality breeding position (higher territory quality or lower relatedness to the partner), or (3) access to extragroup fertilizations. Using the predictions outlined in Table 1, we assessed whether prospectors and floaters obtained these benefits.

*Concept 2a. Obtaining a territory*. We tested if status (philopatric, prospector, or floater) predicted whether an individual obtained a breeding position (binary response variable) by the beginning of the subsequent season (Concept 2a). The first model only included individuals alive at the beginning of the subsequent season. The second model also included individuals that did not survive (included as “did not obtain a breeding position”). We included age (subadult or adult) and sex of each individual as fixed factors. Birds observed in 2014 (for whom subsequent survival/status could not be determined) and birds translocated from the island (Wright et al. 2014) in the year following the focal season were not included.

For individuals that acquired a breeding position, we analyzed whether status, sex, and age predicted the distance between the new and former resident territory. For floaters, the distance was calculated based on their last resident territory (excluding six floaters with unknown origin).

*Concept 2b. Obtaining a better quality breeding position*. Among birds who obtained a breeding position, we analyzed whether status, sex, and age predicted (1) the quality (food availability; square root transformed) of the new territory (excluding three individuals for which territory quality was not measured), or (2) relatedness to the new partner (excluding two individuals that obtained a breeding position but no partner). Pairwise relatedness of breeding pairs (R) was calculated using the Queller and Goodnight (1989) estimation in GenAlEx 6.5 (Peakall and Smouse 2012), using genotypes based on 30 microsatellite loci (see Richardson et al. 2000, Spurgin et al. 2014).

*Concept 2c. Access to extragroup fertilizations*. Subordinates may prospect or float to obtain extrapair fertilizations. Previously, it was shown that male subordinates never obtain extragroup fertilizations (Richardson et al. 2001). Female subordinates, however, may lay an egg in the nest of the breeding pair (Richardson et al. 2001, 2002). To assess whether females prospected to obtain extragroup fertilizations, we first determined whether females prospected in their fertile period (14–1 days before egg‐laying; Komdeur et al. 1999). For the 10 females that did, we could not determine directly whether they had reproduced (we sampled offspring only in territories of two of these), and therefore we determined whether these females helped in incubation, because subordinate females that lay an egg always incubate (Richardson et al. 2003). Egg dumping is absent in this species (Richardson et al. 2001), so we can exclude that females prospect or float to lay an egg in a nonresident territory.

#### Costs of prospecting and floating: condition, survival, and intraspecific attacks

Prospecting or floating may be inhibited by high costs (Concept 4 in Table 1). As adult predation is absent in the Seychelles warbler (Concept 4a), we only assessed physiological costs by testing whether body mass and survival of individuals differed according to their status, and whether prospectors and floaters were attacked by breeders in the territory they intruded.

*Concept 4c1. Body condition*. We determined whether body mass was predicted by status, including variables that may affect body mass in the model (sex, tarsus length, age, time [morning (6:34–10:00), midday (10:00–14:00), afternoon (14:00–19:10)] and month [June–September] of capture). As nestling feeding is costly (Van de Crommenacker et al. 2011a), we excluded helpers caught during or after the nestling period.

*Concept 4c2. Survival*. We assessed whether survival until the subsequent season was predicted by status, age, and sex. Individuals that had obtained a breeding position before the start of the subsequent season were not included, as we did not know how long they had been in that position. We excluded birds from 2014 and translocated individuals (see above).

*Concept 4c3. Intraspecific competition*. On occasion, birds were (opportunistically) caught together while involved in intraspecific chases. In contrast to normal activities, the chases often involve rapid long‐distance flights close to the ground. Consequently, birds involved in chases are regularly caught together in the mist nets (personal observations; see Kingma et al. 2016). Investigating how often individuals were caught together with a resident individual allowed us to infer whether prospectors and floaters were more often attacked than were philopatric subordinates in their resident territory. Whether a subordinate was caught alone or with a resident was the dependent variable and subordinate status (philopatric or prospector or floater) and sex were included as predictors.

#### Predictors of prospecting and floating

Several theories and concepts lead to predictions of how certain factors influence individuals to prospect or float (see Table 1). We assessed whether a suite of factors (see below) predicted whether individuals were (1) strictly philopatric or prospected (excluding floaters), or (2) whether they were philopatric or floated during the season (in a separate model, including prospectors within the class of philopatric individuals). Specifically, we first included the following predictors: age and sex (reflecting competitive ability; concept 5b3 in Table 1), territory quality and group size (reflecting food availability; concepts 4b2 and 4b4, respectively), the number of neighboring territories in the (last) resident territory (reflecting chances of obtaining a nearby breeding position; concept 5b2), and whether both breeders in the (last) resident territory were first‐order relatives of the subordinate (reflecting nepotism and kin‐selected benefits; concept 4b3). Relatedness was assessed based on social pedigree data because nepotism and helping behavior are probably affected by familiarity between individuals (Komdeur et al. 1996) rather than genetic relatedness per se (which may differ because of extrapair fertilizations; Richardson et al. 2004).

In a second set of models, we included the same predictors but instead of relatedness to both breeders, we included whether individuals were related to the opposite‐sex breeder, as proxy for potential mating opportunities (without inbreeding) in their resident territory (concept 5c2; Kingma et al. 2011). The model with floaters did not converge and we therefore performed this model without the random effect “year.”

In the third and fourth models we included the same predictors as in the original model, and also added as predictors (1) whether subordinates helped during the season (because benefits of help may drive delayed dispersal; concept 5d1) or (2) residual body mass in their resident territory (to assess whether prospecting is condition dependent: concept 4c4), respectively. For these models we used subsets of data because we did not have complete data about helping decisions or body mass for some individuals. These analyses were only performed for individuals that had a resident territory; thus not for floaters as these could not help and did not have a body mass measure in a resident territory. Residual body condition measures were obtained from a model with body mass as response variable and tarsus length, month and time of capture as independent variables, and year as a random variable (see Table 3). As only five males were caught in their resident territory and observed prospecting during the same season, we restricted the model with body mass as predictor to females.

## Results

Overall, we identified prospecting trips by 54 of 390 subordinates (14%) and assigned another 38 individuals as floaters (9% of the overall total of 428 individuals). At least 42 of the 54 prospectors returned to their resident territory after a prospecting trip (and even those individuals not seen back in the resident territory may well have returned after the end of the fieldwork period). The average (±SE) distance between the territory where prospectors were observed and their resident territory was 5.3 ± 0.3 territories (range: 3–12). The maximum distance between territories on the island was 16 territories.

### BENEFITS OF PHILOPATRY, PROSPECTING, AND FLOATING: TERRITORY ACQUISITION AND EXTRAGROUP FERTILIZATIONS

**Figure 1:**
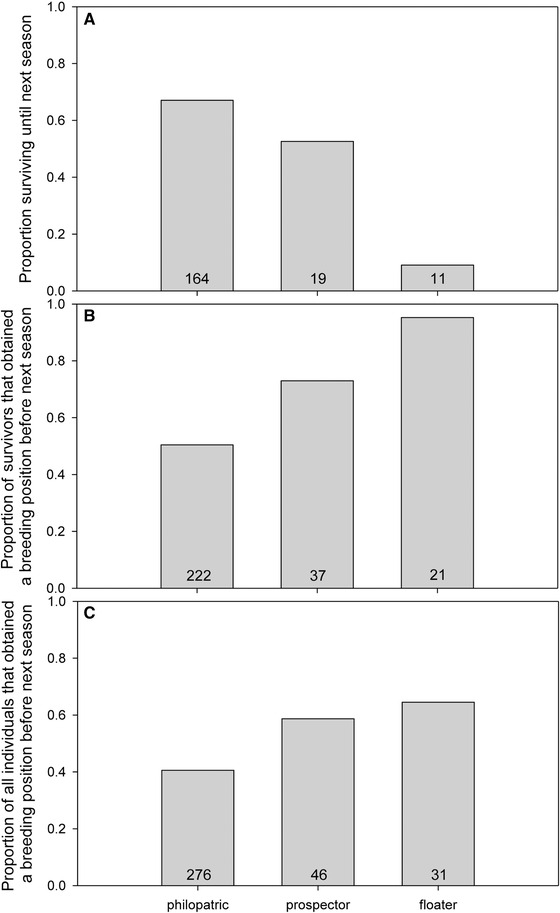
The effect of whether individual Seychelles warblers were “strictly philopatric,” “prospecting while philopatric,” or “floating” on (A) survival (only individuals that did not obtain a breeding position before the subsequent breeding season) or (B and C) obtaining a breeding position before the subsequent breeding season. (B) Only includes individuals that survived and (C) includes all individuals (including those that died). Numbers of individuals are shown. Test statistics are provided in Table 2.

*Concept 2a. Obtaining a territory*. Prospectors and floaters that survived until the next season were significantly more likely to have obtained a breeding position by the next season (73% and 95% of individuals, respectively) compared to the surviving strictly philopatric subordinate birds (50%; Table 2B, Fig. 1B). Although still significant (Table 2C), this difference became smaller when individuals that did not survive until the next season were included as “not having obtained a breeding position” (Fig. 1C), because prospectors and floater were less likely to survive (see below and Fig. 1A).

**Table 2:** The effect of sex, age, and status (whether a bird prospected or floated) on whether subordinate Seychelles warblers (A) survived until the next season (only individuals that did not get a breeding position), and (B and C) had a breeding position at the beginning of the next season ((B) only includes individuals that survived and (C) includes all individuals)

	β	SE	*z*	*P*
**(A) Subordinate survived until next season?**				
*Intercept*	0.746	0.221	3.382	**<0.001**
Sex^1^	−0.344	0.342	−1.006	0.315
Age^2^	0.155	0.363	0.426	0.670
Status^3^				
Prospector	−0.595	0.506	−1.176	0.240
Floater	−2.897	1.081	−2.681	**0.007**
**(B) Survivors—gaining breeding position before next season?**				
*Intercept*	0.066	0.176	0.376	0.707
Sex^1^	0.519	0.273	1.904	0.057
Age^2^	−0.191	0.290	−0.656	0.512
Status^3^				
Prospector	0.967	0.400	2.419	**0.016**
Floater	2.860	1.039	2.752	**0.006**
**(C) All individuals—gaining breeding position before next season?**				
*Intercept*	−0.358	0.133	−2.691	**0.007**
Sex^1^	0.349	0.252	1.383	0.167
Age^2^	−0.191	0.253	−0.754	0.451
Status^3^				
Prospector	0.723	0.325	2.225	**0.026**
Floater	0.940	0.403	2.332	**0.020**

See Figure 1 for sample sizes and graphical representation.

^1^Males relative to females.

^2^Subadults (5–12 months old) relative to adult subordinates (1–2 years old).

^3^Prospectors and floaters relative to philopatric individuals.

Prospectors (median distance to obtained territory = 4 territories, range = 1–13, *n* = 29; β = 0.317 ± 0.102, *z* = 3.123, *P* = 0.002) and floaters (median = 4, range = 2–10, *n* = 16; β = 0.444 ± 0.126, *z* = 3.530, *P* < 0.001) that obtained a breeding position obtained this position further away from their former territory than did philopatric individuals (median = 2, range = 0–11, *n* = 125). Overall, males obtained a position closer to their original territory (median = 2, range = 0–11, *n* = 84) than females (median = 5, range = 0–13, *n* = 86; β = −0.762 ± 0.092, *z* = −8.262, *P* < 0.001), and adult subordinates obtained a position closer (median = 2, range = 0–9, *n* = 58) than subadults (median = 3, range = 0–13, *n* = 112; β = −0.268 ± 0.093, *z* = −2.871, *P* = 0.004). Of the 125 philopatric individuals, 14 (11%) obtained their breeding position by inheriting their resident territory before the subsequent season, but no prospectors did.

*Concept 2b. Obtaining a better quality breeding position*. The quality of the territory where individuals obtained a breeding position was not different between strictly philopatric individuals (*n* = 124) and prospectors (*n* = 27; β = −10.752 ± 8.533, *t* = −1.260, *P* = 0.209) or between philopatric individuals and floaters (*n* = 22; β = −11.635 ± 9.374, *t* = −1.241, *P* = 0.216), and was not predicted by sex (males: β = −2.185 ± 6.163, *t* = −0.355, *P* = 0.723) or age (adults; β = 1.519 ± 6.669, *t* = 0.228, *P* = 0.820).

Prospectors, but not floaters, who obtained a breeding position before the beginning of the next season were less related to their new partner than philopatric individuals who gained a breeding position (prospectors: R = −0.056 ± 0.036, *n* = 28, β = −0.100 ± 0.045, *t* = −2.215, *P* = 0.028; floaters: R = 0.022 ± 0.046, *n* = 21, β = −0.021 ± 0.051, *t* = −0.423, *P* = 0.673; philopatric individuals: R = 0.043 ± 0.020, *n* = 125). Relatedness to the new partner was not predicted by sex (males; β = 0.013 ± 0.034, *t* = 0.384, *P* = 0.702) or age (adults; β = −0.045 ± 0.034, *t* = −1.302, *P* = 0.195).

*Concept 2c. Access to extragroup fertilizations*. The majority of prospecting females (74% of 39) prospected outside their fertile period. The remaining 10 females prospected less than 2 weeks before egg‐laying commenced in their resident territory. None of the seven prospecting females for which we conducted an incubation watch in their resident territories was observed incubating, indicating that none of these females had laid an egg after their prospecting trip (reproducing subordinates always incubate; Richardson et al. 2001, 2003).

### COSTS OF PROSPECTING AND FLOATING: CONDITION, SURVIVAL, AND INTRASPECIFIC ATTACKS

*Concept 4c1. Body condition*. Correcting for time and month of capture, sex, tarsus length, and age, individuals caught while prospecting had lower body mass than individuals caught in their resident territory; floaters also had a slightly lower body mass than philopatric individuals but this effect was not significant (Table 3).

**Table 3:** The effect of status at catching (whether a bird prospected or floated), age, tarsus length, and the time and month of capture on 237 subordinate Seychelles warblers’ body mass

	β	SE	*t*	*P*
*Intercept*	−1.242	1.191	−1.043	0.298
Moment of catch^1^				
While prospecting (*n* = 20)	−0.532	0.169	−3.152	**0.002**
While floating (*n* = 23)	−0.235	0.159	−1.475	0.142
Age^2^	0.272	0.134	2.037	**0.043**
Tarsus length	0.628	0.047	13.336	**<0.001**
Time^3^				
Midday	0.099	0.125	0.790	0.430
Afternoon	0.603	0.121	4.967	**<0.001**
Month^4^				
July	0.520	0.133	3.903	**<0.001**
August	0.917	0.140	6.536	**<0.001**
September	0.770	0.169	4.560	**<0.001**

^1^Relative to 194 birds caught in their resident territory.

^2^Subadult (5–12 months old) relative to adult (1–2 years old) subordinates.

^3^Relative to morning catches.

^4^Relative to catches in June.

*Concept 4c2. Survival*. Floaters, but not prospectors, had a lower chance of survival until the next season than did philopatric individuals (9%, 53%, and 67% of floaters, prospectors, and philopatric individuals that did not obtain a breeding position survived, respectively; see Table 2A, Fig. 1A).

*Concept 4c3. Intraspecific competition*. Prospectors (three of 20 catches (15%); β = 1.819 ± 0.783, *z* = 2.322, *P* = 0.020) and floaters (four of 23 (17%); β = 1.465 ± 0.664, *z* = 2.207, *P* = 0.027) were substantially more often caught together with an individual resident to the intruded territory than philopatric individuals (5% of 194). Overall, males (10% of 113) were more often caught with another individual than females (3% of 124; β = 1.689 ± 0.638, *z* = 2.648, *P* = 0.008).

### PREDICTORS OF PROSPECTING AND FLOATING

Males (9% of 162) were less likely to prospect than females (19% of 175; Table 4). Whether individuals prospected during the season was not predicted by age, group size, whether both breeders were related, or the number of neighboring territories, but there was a nonsignificant tendency (*P* = 0.083; Table 4) for individuals in better quality territories to be more likely to prospect. Whether the opposite‐sex breeder in the resident territory was related did not predict whether a subordinate prospected (14% of 271 individuals with a related, and 14% of 66 with an unrelated, opposite‐sex breeder prospected; Table 4). The likelihood that female subordinates prospected was also not predicted by body mass or by whether the individual helped or not during the season (13% of 85 helpers and 15% of 65 nonhelpers prospected; Table 4).

**Table 4:** The effect of sex and age of subordinate Seychelles warblers and territory quality, group size, the number of neighboring territories, and whether both parents were still present on the probability that subordinates Seychelles warblers were observed (A) prospecting (total *n* = 337) or (B) floating (*n* = 361)

	(A) Prospecting				(B) Floating			
	β	SE	*z*	*P*	β	SE	*z*	*P*
*Intercept*	1.422	0.191	−7.445	**<0.001**	−4.548	1.030	−4.416	**<0.001**
Sex^1^	−0.936	0.339	−2.763	**0.006**	0.049	0.455	0.107	0.914
Age^2^	0.380	0.339	1.119	0.263	0.356	0.470	0.757	0.449
Territory quality (log10)^3^	0.792	0.456	1.735	*0.083* 3	−0.550	0.692	−0.795	0.427
Group size	−0.075	0.194	−0.389	0.697	0.681	0.236	2.890	**0.004**
Number of neighboring territories	−0.092	0.104	−0.881	0.378	0.018	0.144	0.127	0.899
Both parents present?^4^	0.269	0.372	0.723	0.470	−1.528	0.469	−3.261	**0.001**
					
Opposite‐sex breeder related?^5^	0.110	0.403	0.274	0.784	−0.265	0.533	−0.497	0.619
Helping^6^	−0.375	0.485	−0.772	0.440				
				
Body condition 7	−0.064	0.467	−0.137	0.891				

The effects of whether the opposite‐sex breeder was related^5^, whether subordinates helped^6^, and body condition (females only^7^) were tested in separate models.

^1^Males relative to females.

^2^Subadult (5–12 months old) relative to adult (1–2 years old) subordinates.

^3^Territory quality data from 2005 were estimated based on average territory quality of 2004 and 2006 (see Methods section). The effect is slightly larger when estimated data from 2005 were excluded (*n* = 312, β = 0.842 ± 0.461, *z* = 1.829, *P* = 0.067).

^4^Subordinates living with related breeders, relative to birds in territories where the subordinate is unrelated to one or both breeders.

^5^Opposite‐sex breeder being first‐order relatives, relative to when the opposite‐sex breeder was unrelated.

^6^Birds that helped during the season in brood care relative to individuals that did not help. Note that these results were obtained in a subset model with 150 individuals for which we had information on helping behavior. Data on helping behavior were not available for floaters.

^7^Residual condition; body mass corrected for significant effects of tarsus length, and month and time of capture (see Table 4). Note that the results were obtained from a subset model with 78 individuals, only females, which were caught to assess body condition. Data on body condition in a resident territory were not available for floaters.

**Figure 2:**
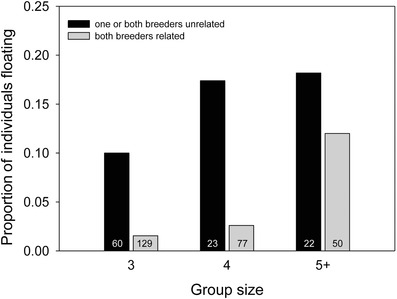
The effect of group size and whether both breeders were related (first‐order relative) versus at least one unrelated on the probability that subordinate Seychelles warblers become floaters. Numbers denote sample size. Note that group sizes of 5 and 6 were grouped for graphical purpose, as group sizes of six individuals are rare (*n* = 12). Test statistics are provided in Table 4.

Females and males were equally likely to float and floating was not related to age, territory quality, or number of neighboring territories (Table 4). The likelihood that subordinates floated was, however, significantly higher when one or both parents in their former resident territory were replaced by an unrelated breeder and when that former group contained more individuals (Table 4, Fig. 2). Whether the opposite‐sex breeder was related did not predict whether individuals became floaters (Table 4).

## Discussion

Our comprehensive investigation of different routes to independent breeding for subordinate Seychelles warblers shows that their associated costs and benefits differ. Opportunities for independent breeding are high for prospectors and floaters compared to those for strictly philopatric individuals. However, the costs of prospecting (lower body condition) and floating (lower survival) are also higher. These elevated costs are especially apparent for floaters, providing one adaptive explanation for why individuals remain philopatric. The high survival costs of floating, in combination with the ability to obtain comparable benefits through prospecting, suggests that remaining as a subordinate in a territory with occasional prospecting may represent the best strategy when opportunities for independent breeding are limited. We discuss these and other results in detail below and highlight the importance of considering different routes to independent breeding to understand delayed dispersal in social vertebrates.

### THE COSTS AND BENEFITS OF PHILOPATRY, PROSPECTING, AND FLOATING

Subordinate individuals in social species may gain survival and reproductive benefits by prospecting or floating, compared to remaining strictly philopatric (see Table 1) if these behaviors facilitate access to refuge, food, or extragroup fertilizations. However, our analyses suggest that this is not the case in the Seychelles warbler: (1) floaters and prospectors are regularly attacked by conspecifics; (2) are in worse condition than philopatric individuals; and (3) extraterritorial forays do not seem to facilitate acquisition of extragroup fertilizations (concurring with the fact that most subordinate females prospected outside their fertile period and subordinate males rarely obtain extragroup paternity; Richardson et al. 2001). Instead, the main benefit for prospectors and floaters appears to be a much higher likelihood of obtaining an independent breeding position compared to strictly philopatric individuals (73% and 95% vs. 50%). This difference is likely to be a conservative estimate as we must have missed prospecting trips (e.g., outside the fieldwork season): some birds assigned as “strictly philopatric” obtained a position more than two territories from their resident territory and must, by definition, at some point have engaged in prospecting trips. Prospectors and floaters also obtained a position further away from their former resident territory than strictly philopatric individuals that were not observed prospecting or floating, and although this did not affect the quality of the obtained territory in terms of food availability, prospectors (but not floaters; see below) were less related to their partner in their new territory than philopatric individuals. This lower relatedness may subsequently reduce the negative consequences of inbreeding observed in this system (Richardson et al. 2004, Bebbington et al. 2016). Although we could not determine the duration and frequency of prospecting trips, it appears likely that prospecting individuals improve their chances of discovering a (suitable) vacancy, or gain greater knowledge about potential future vacancies (e.g., by assessing health of breeders or local density of competitors; Bocedi et al. 2012; Delgado et al. 2014). Either way, floaters and prospectors showed the same improved likelihood of eventual dispersal, suggesting that the underlying mechanism is similar for floaters and prospectors. Thus, there appear to be substantial functional benefits of prospecting and floating in the form of improved breeding opportunities, at least in the short term. Future studies investigating reproductive performance are now important to determine the long‐term fitness consequences of different routes to breeding.

On the other hand, prospecting and floating were associated with substantial costs. Individuals captured during prospecting or floating had considerably lower body mass than philopatric subordinates. This difference was not statistically significant for floaters, possibly due to differential mortality of floaters in poor condition. Apart from potential energetic costs of increased movement per se, a plausible explanation for reduced body mass during extraterritorial forays is that extraterritorial forays often result in aggressive interactions with conspecifics (Kingma et al. 2016). Although we could not show this directly, such interactions could lead to reduced food intake of prospectors and floaters, compared to philopatric subordinates who may have undisturbed access to resources because of acceptance by breeders (Griesser et al. 2006; Eikenaar et al. 2007). Indeed, the importance of a resident “safe‐haven” territory in the Seychelles warbler is illustrated by a considerably lower survival probability for floaters than for prospectors who return to their resident territory. It is possible that floating in the Seychelles warbler is relative costly compared to floating in “open” populations in several other species, because this closed and saturated population provides only limited suitable possibilities for floaters to take refuge and escape from attacks by conspecifics. However, in several social species, nepotistic benefits (access to food and refuge against conspecifics and predators) are cited as one of the main benefits of delayed dispersal (Ekman et al. 1994; Covas and Griesser 2007). As such, although predation on adult Seychelles warblers is absent, in species where extraterritorial movement induces higher predation risk, nepotistic protection is probably an even stronger selection pressure for delayed dispersal (Griesser 2003). Interestingly, we found that some philopatric Seychelles warblers are unrelated to one (13%) or both (18%) breeders in the territory. However, reduced relatedness did not affect the likelihood that individuals aimed to leave voluntarily (i.e., prospected). This suggests that nepotism and kin benefits cannot fully explain why individuals stay in this system. One explanation may be that unrelated subordinates are allowed to remain in the territory when they are beneficial for breeders, for example through territory defense or helping at the nest, but this needs to be explored in more detail. Having unrelated breeders and larger groups in a resident territory did not predict voluntary prospecting by subordinates, but did predict floating. Consequently, we suggest that floaters may be evicted from their resident territory (see below) if they are less related to breeders and/or when groups are larger (with higher group size resulting in lower per capita food availability; Brouwer et al. 2006) because they impose a larger cost to breeders (see also Ekman and Griesser 2002).

To make inferences about whether the energetic benefits of philopatry (e.g., nepotism) drive delayed dispersal, additional benefits like the reproductive benefits of philopatry also need to be explored. Subordinate Seychelles warblers can obtain direct, indirect, and future reproductive benefits in their resident territory, but this does not seem to explain delayed dispersal. First, subordinates can inherit their territory (8% of positions) or gain a breeding position in a nearby territory (44% one or two territories distance; Table 1), but our predictors for such benefits (relatedness to the opposite‐sex breeder and local breeding density) did not predict whether individuals prospected or floated. Second, young subordinate females are more likely to prospect despite gaining significantly more parentage in their resident territory than males (Richardson et al. 2002, 2003). Third, we find that subordinates are more likely to float when they are unrelated to one or both breeders. This is in line with our predictions that subordinates who *are* related to breeders should be more inclined to stay and obtain indirect benefits in the resident territory. However, the lack of evidence for a similar pattern in prospectors (i.e., attempts to leave), and the fact that whether subordinates helped did not predict whether individuals prospected, suggests that potential indirect benefits of helping do not affect subordinates’ motivation to stay or leave. Therefore, our results suggest that attempted dispersal by prospecting is costly, and that delayed dispersal (at least by birds younger than 2 years old as included here) is, at least partly, driven by the energetic, but not reproductive, benefits of philopatry. Such benefits of philopatry appear to be facilitated by acceptance of subordinates by breeders: a key prerequisite for philopatry and group formation to occur in this species (or any species) in the first place.

Our results highlight a trade‐off between the costs and benefits of prospecting: if only individuals in good condition can overcome the costs associated with prospecting, then prospecting will be condition dependent (Zöttl et al. 2013). Although we did not find evidence that body condition predicted prospecting, the result of this analysis may be misleading. Apart from the fact that we may have missed some prospecting trips, the decision to prospect may be based on condition just before leaving the natal territory, and our single measurement during the season probably lacks sufficient resolution to detect an effect. We observed prospecting mainly in periods of high food availability (see Supporting Information Appendix), and there was a tendency for individuals from high‐quality territories to be more likely to prospect. Thus, subordinates seem, at least to some degree, to be physiologically constrained in engaging in prospecting. However, when there are sufficient benefits in a resident territory (acceptance by breeders, sufficient food availability) they can remain philopatric, or return after prospecting, to wait for future dispersal opportunities. Together, these results highlight that ecological constraints and survival benefits of philopatry can be considered as two sides of the same coin (i.e., the relative cost of extraterritorial movement), and that delayed dispersal, often a prerequisite for cooperative breeding (Cockburn 1998), provides a better alternative than floating when breeding opportunities are limited, especially if philopatric individuals can also prospect.

### DELAYED DISPERSAL: ASSESSING DIFFERENT ROUTES TO INDEPENDENT BREEDING

Our results confirm that considering the different potential routes to independent breeding is important to understand delayed dispersal. First, the magnitude of the costs and benefits of the different routes greatly differ, largely depending on whether individuals can return (prospectors) or not (floaters) to their resident territory after extraterritorial forays. These differences are important because they affect the relative benefits of delayed dispersal compared to other routes of dispersal. Failure to consider prospecting may have considerable implications for parameter estimates in models of delayed dispersal (e.g., in Koenig et al. 1992; Kokko and Ekman 2002) and, given that prospecting by philopatric individuals yields similar benefits to floating, ignoring prospecting may lead to erroneous conclusions about the adaptive benefits of floating. Moreover, although floaters are more likely to obtain a territory in the short term, philopatric individuals have higher survival rates and may therefore obtain a breeding position later in life, whereas unsuccessful floaters die in almost all cases (see Fig. 1 and below). For example, the likelihood that individuals that had not obtained a position in a given year would do so the next year (estimated based on multiplying the probabilities of survival and obtaining a position (see Fig. 1) and assuming individuals adopt the same strategy) is only 9% for floaters (9% survival probability multiplied by 95% probability of obtaining a breeding position; see Fig. 1), but 39% (53% × 73%) and 34% (67% × 50%) for prospectors and strictly philopatric individuals, respectively. Therefore, although effects on life‐time fitness remain to be tested, remaining philopatric (with the additional option of prospecting if condition allows) may be a safer strategy than floating. A previous study on the Seychelles warbler (Hammers et al. 2013) showed that individuals that breed later in life have similar reproductive tenure compared to those breeding earlier in their life, suggesting that delayed dispersal may not be as costly as it first appears. However, Hammers et al. (2013) focused on individuals that reached old age *and* obtained a breeding position, but did not consider individuals that died as floaters or while philopatric (and thus forewent independent breeding altogether). We argue that studies on the fitness effects of delayed dispersal should account for the fact that individuals may die before obtaining an independent breeding position and that mortality rates may differ between individuals using different strategies to obtain a breeding position.

Considering the different routes to breeding enables explicit testing of the intrinsic, environmental, and social factors that drive an individual's dispersal decision. This permits investigation of the proximate selective forces behind delayed dispersal, rather than those behind independent breeding of a subset of individuals that obtained a breeding position (Brown 1987; Ekman 2006). For example, our data suggest that floaters are often evicted, whereas prospectors leave voluntarily. Therefore, different proximate mechanisms may underlie ultimate dispersal through these routes. If considering only floaters, one could conclude that improved food availability in smaller groups drives delayed dispersal. However, as group size does not predict prospecting, we argue that floating—which is practiced more often by individuals from larger groups—may instead be a consequence of eviction (see also Pasinelli and Walters 2002). Similarly, whether dispersal attempts are forced or voluntary may also complicate the interpretation of studies showing that delayed independent breeding leads to higher reproductive success (e.g., Hawn et al. 2007; Guinapp and Merilä 2011): if early‐dispersing individuals are evicted and cannot return to a resident territory, such individuals are probably more likely to accept lower quality positions (as suggested by the result that prospectors but not floaters, are less related to their future partner; see also Fig. 1 in Koenig et al. 1992). Future studies should estimate the life‐time fitness of individuals with different dispersal strategies or routes to independent breeding, while considering mortality during dispersal and the proximate factors and mechanisms that may underlie dispersal.

## Conclusions

Given that prospecting and floating can be beneficial compared to strict philopatry, exploring the individual characteristics and the social and ecological factors that determine when individuals engage in these behaviors can help make inferences about the evolution of delayed dispersal. We provided a framework to test the suite of factors that are predicted by the ecological‐constraints and benefits‐of‐philopatry hypotheses to explain prospecting and floating in social species (Table 1). We hope that this helps future empirical and theoretical studies to further unravel the importance of simultaneously assessing the different routes to independent breeding, for example by including long‐term reproductive success data, to understand delayed dispersal in social animals.

## Supporting information

Additional Supporting Information may be found in the online version of this article at the publisher's website:

**Figure S1**. inferences about prospecting behavior in relation to monthly food abundance.
