# Supplementary material for: Delayed dispersal and the costs and benefits of different routes to independent breeding in a cooperatively breeding bird
Source: Evolution. 2016 Oct 6;70(11):2595–610. doi: 10.1111/evo.13071 (PMC5132126; doi:10.1111/evo.13071)
Supplement: Supplementary file 1 — Figure S1. inferences about prospecting behavior in relation to monthly food abundance. [file EVO-70-2595-s001.docx]

**Delayed dispersal and the costs and benefits of different routes to independent breeding in a cooperatively breeding bird**

Sjouke A. Kingma, Kat Bebbington, Martijn Hammers, David S. Richardson, Jan Komdeur

**SUPPLEMENTARY MATERIAL**

To make inference about whether individuals prospected mainly during periods of high food abundance (i.e. when the energetic costs of prospecting may be lower), we first calculated average monthly arthropod abundance during the main breeding season (June till September) (see Komdeur (1992), Van de Crommenacker (2011) and main text for details). Second, for each year during the main breeding season we determined how many days we had performed field-work and how many prospectors were observed or caught each month in order to calculate for each month the ‘average number of prospectors per day’. Most prospectors were observed in July and August (Fig. S1b), the period that also most arthropods were present (Fig. S1a).

**Figure S1.** (a) The average number of arthropods from June – September 2003-2014. Numbers denote number of years in which arthropods were counted for each month. (b) Average number of prospectors observed and caught per day in each month. Numbers denote number of days.

#

# Supplementary References

# Komdeur, J. 1992. Importance of habitat saturation and territory quality for evolution of cooperative breeding in the Seychelles warbler. Nature 358:493–495.

# Van de Crommenacker, J., J. Komdeur, and D. S. Richardson. 2011. Assessing the cost of helping: the roles of body condition and oxidative balance in the Seychelles Warbler (*Acrocephalus sechellensis*). PLoS ONE 6:e26423.
